# Achieving BMI <25 kg/m^2^ was associated with reduced predicted risk of atherosclerotic cardiovascular disease in people with obesity or overweight on tirzepatide or placebo: a post hoc analysis of SURMOUNT-1, -3, and -CN

**DOI:** 10.1016/j.eclinm.2025.103722

**Published:** 2025-12-27

**Authors:** Lixin Guo, Shan Ding, Weihao Wang, Hanxi Zhang, Shaojun Dai, Chengwei Li, Yuan Yuan, Adam Stefanski, Irina Jouravskaya, Tammy D. Forrester

**Affiliations:** a Department of Endocrinology, Beijing Hospital, National Center of Gerontology; Institute of Geriatric Medicine, Chinese Academy of Medical Sciences, China; b Eli Lilly and Company, Suzhou, China; c Eli Lilly and Company, Indianapolis, IN, USA

**Keywords:** Obesity, Weight reduction, ASCVD, Tirzepatide

## Abstract

**Background:**

Effective weight reduction interventions may significantly reduce obesity-related atherosclerotic cardiovascular disease (ASCVD) risk. However, evidence on the association between optimal body mass index (BMI) target and long-term ASCVD risk was limited.

**Methods:**

Pooled individual-level data from participants with obesity/overweight randomized to tirzepatide or placebo in SURMOUNT-1 (December 2019–April 2022; NCT04184622), -3 (March 2021–April 2023; NCT04657016), and -CN trials (September 2021–December 2022; NCT05024032) were analyzed. Ten-year ASCVD risk was calculated using the American College of Cardiology/American Heart Association Pooled Cohort Equations. A mixed model for repeated measures analysis was used to compare percent change in ASCVD risk from baseline by achieved BMI group (<25 kg/m^2^, ≥25 kg/m^2^) at trial end, with terms including achieved BMI group, time point, achieved-BMI-group-by-time-point interaction, and baseline covariates.

**Findings:**

Among 2691 participants included, 495 (18.4%) achieved BMI <25 kg/m^2^ at trial end. In addition to a significantly higher proportion being treated with tirzepatide (98.2% vs 66.8%), the BMI <25 kg/m^2^ group also had a higher proportion of females and lower mean BMI at baseline compared with the BMI ≥25 kg/m^2^ group (*P* < 0.001 for all). After adjusting for baseline covariates, relative to baseline, participants achieving BMI <25 kg/m^2^ had a significantly greater percent reduction in predicted ASCVD risk (39.4% vs 10.6%, *P* < 0.001) compared to the BMI ≥25 kg/m^2^ group. Among those with baseline intermediate-to-high ASCVD risk, reduction remained greater in the BMI <25 kg/m^2^ group (25.6%) than the BMI ≥25 kg/m^2^ group (9.0%; *P* < 0.001). Significantly greater improvements were also observed in blood pressure and lipids for the BMI <25 kg/m^2^ group (*P* < 0.001).

**Interpretation:**

Achieving BMI <25 kg/m^2^, primarily with tirzepatide, was associated with a significantly greater 10-year ASCVD risk reduction compared with those whose BMI remained at 25 kg/m^2^ or greater. These findings suggest potential cardiovascular benefits associated with targeting BMI <25 kg/m^2^ in the long-term weight management.

**Funding:**

This study was funded by 10.13039/100004312Eli Lilly and Company.

**Research in context**

**Evidence before this study**

Obesity is a major global health concern, significantly increasing the risk of atherosclerotic cardiovascular disease (ASCVD). Current guidelines mostly recommend 5–15% weight reduction to prevent complications, but a clear weight management target that is suitable for the long term is less clear. Long-term stringent BMI control (e.g., BMI <25 kg/m^2^) may have become increasingly feasible with the assistance of novel pharmacotherapies such as the glucose-dependent insulinotropic polypeptide and glucagon-like peptide-1 receptor agonist. A search of the PubMed for studies evaluating the association between long-term optimal BMI target and ASCVD risk (from inception to October 2025, using search terms obesity, overweight, BMI, cardiovascular disease, ASCVD, and weight reduction/loss) indicated that such evidence is lacking.

**Added value of this study**

To our knowledge, this is the first study to investigate the association between achieving BMI <25 kg/m^2^ and long-term predicted ASCVD risk among individuals with obesity or overweight. This study found that participants who achieved BMI <25 kg/m^2^ after 52 or 72 weeks of tirzepatide or placebo treatment had significantly greater reductions in 10-year predicted ASCVD risk compared with those who did not. Additionally, participants achieving BMI <25 kg/m^2^ exhibited significantly greater improvements in key cardiometabolic factors including systolic and diastolic blood pressure, low-density lipoprotein cholesterol, and non-high-density lipoprotein cholesterol. These findings suggested that targeting weight reduction at BMI <25 kg/m^2^ might provide potential cardiovascular benefits in the long term.

**Implications of all the available evidence**

As more data with longer follow-ups become available, future studies are warranted to evaluate the association between BMI <25 kg/m^2^ and observed incident ASCVD risk. Future clinical guidelines might consider evaluating benefits and risks of targeting BMI <25 kg/m^2^ for individuals with obesity or overweight, particularly for those at a high risk of cardiovascular events.

## Introduction

Obesity, a chronic relapsing disease with escalating global prevalence,^1^ is projected to affect over 1 billion individuals by 2025 according to the World Obesity Federation projections.^2^ The major comorbidities associated with obesity include type 2 diabetes (T2D), dyslipidemia, hypertension, and cardiovascular disease (CVD).^3^^,^^4^ Epidemiologic evidence demonstrated that individuals with obesity had an increased risk of atherosclerotic CVD (ASCVD) by more than two-fold compared with those without obesity,^5^ with each 5-unit increase in body mass index (BMI) associated with approximately 30% higher ASCVD risk.^6^ Robust evidence exists supporting that effective weight reduction interventions may significantly reduce obesity-related morbidity and mortality, particularly ASCVD outcomes.^7^

The World Health Organization uses BMI to define overweight (BMI ≥25 kg/m^2^) and obesity (BMI ≥30 kg/m^2^),^8^ whereas some expert guidelines recommend lower BMI thresholds in defining obesity in Asian populations due to the increased risk of cardiometabolic diseases starting at lower BMI levels.^9^ Recent studies have shown that individuals with BMI ≥25 kg/m^2^ have an increased risk of developing at least one obesity-related comorbidity.^10^ To prevent complications and to optimize clinical outcomes, a weight reduction target of 5%–15% is generally recommended by current guidelines. The American Heart Association/American College Cardiology/The obesity Society (AHA/ACC/TOS) clinical practice guidelines recommend an initial goal of 5–10% body weight reduction within 6 months as the primary therapeutic objective.^11^ The American Association of Clinical Endocrinologists/American College of Endocrinology (AACE/ACE) guidelines recommend a complication-specific target ranging from 5% to 15% weight reduction for chronic disease prevention.^12^ Chinese clinical guidelines on long-term weight management recommend an initial target of 5–10% or 10–15% weight reduction, depending on individuals’ age and comorbidity profile.^13^ Although obesity is a chronic condition requiring long-term management, consensus on a clear weight reduction target in the long term (e.g., BMI <25 kg/m^2^), which is similar to the recommended A1C target of <7.0% in diabetes treatment,^14^ has not been reached. It has been well known that sustainable weight reduction is highly challenging,^15^ and evidence has been limited on the clinical benefits of stringent BMI control.

Novel anti-obesity pharmacotherapies, such as glucagon-like peptide-1 (GLP-1) receptor agonists and dual receptor agonist of the glucose-dependent insulinotropic polypeptide (GIP) and GLP-1 receptors, have emerged as promising therapeutic options, demonstrating significant efficacy in promoting sustained weight reduction.^16^ Their multimodal mechanisms, including targeting appetite regulation, glycemic control, and adipose metabolism, enhance the feasibility of achieving clinically meaningful weight reduction. In the phase 3 SURMOUNT-1 randomized controlled trial (NCT04184622), non-diabetic individuals with obesity or overweight randomized to tirzepatide achieved a mean body weight reduction of up to 20.9% at week 72, with up to 90.9% and up to 56.7% attaining ≥5% and ≥20% weight reduction, respectively. Similar efficacy results in weight reduction have been shown in additional trials such as SURMOUNT-3 (NCT04657016) and -CN (NCT05024032). The availability of modern treatment options has potentially made stringent BMI control at <25 kg/m^2^ a more attainable weight management target for some individuals with obesity or overweight.

To shed light on the association between stringent BMI control and ASCVD risk among individuals with obesity or overweight and inform optimal weight management strategies in the long term, this study analyzed data pooled from the SURMOUNT-1, -3, and -CN trials to assess changes in the predicted 10-year ASCVD risk among participants who achieved BMI <25 kg/m^2^.

## Methods

### Trial design and analysis population

To include a large and diverse population for increased generalizability, this post hoc analysis used the Efficacy Analysis Sets (EASs) from the SURMOUNT-1, -3, and -CN (NCT04184622, NCT04657016, and NCT05024032) trials, which all enrolled participants with obesity (defined as BMI ≥30 kg/m^2^ in SURMOUNT-1 and -3 and as BMI ≥28 kg/m^2^ in SURMOUNT-CN) or overweight (defined as BMI ≥25 kg/m^2^ to <30 kg/m^2^ in SURMOUNT-1 and -3 and as BMI ≥24 kg/m^2^ to <28 kg/m^2^ in SURMOUNT-CN) without diabetes.^17^, ^18^, ^19^ The EAS for each trial was derived from the respective modified intent-to-treat (mITT) population, defined as all randomized participants receiving ≥1 dose of study medication, and included data captured during treatment period (up to last dose date + 7 days). Detailed study design for SURMOUNT-1, -3, and -CN have been previously described. In brief, SURMOUNT-1, -3, and -CN were all phase 3, multicenter, randomized, double-blind, placebo-controlled trials where participants were randomized to receive tirzepatide or placebo for 72 weeks (SURMOUNT-1 and -3) or 52 weeks (SURMOUNT-CN). SURMOUNT-1 (n = 2539) and -3 (n = 579) both enrolled patients from multiple countries, whereas SURMOUNT-CN (n = 210) enrolled patients exclusively from China.

Participants were included in the post hoc analysis if they had BMI ≥25 kg/m^2^ at baseline. In addition, included participants had to have non-missing predicted ASCVD risk as cacluated using the ACC/AHA Pooled Cohort Equations (PCE) at baseline and at ≥1 follow-up time point (week 24 and trial end). More details on the ASCVD risk calculation are described later in the section.

### Ethics

The original SURMOUNT-1, -3, and -CN trials adhered to the principles of the Declaration of Helsinki and Good Clinical Practice guidelines. All protocols were approved by independent ethics committees or institutional review boards (Supplementary Tables S1–3), and all the participants provided written informed consent for the SURMOUNT-1, -3, and -CN trials before participation.

### Ten-year predicted ASCVD risk

The 10-year risk of incident ASCVD was calculated among participants without a history of ASCVD at baseline using the ACC/AHA PCE, with predictors including sex, race, age, total cholesterol, high-density lipoprotein, SBP, hypertension treatment, type 2 diabetes, and current smoking status.^20^ The prediction model was originally developed from community-based cohorts of African American and White participants aged 40–79 years.^21^ The ACC/AHA PCE may offer advantages over conventional Cox proportional hazards models as a standardized tool for ASCVD risk prediction, and has demonstrated validity in real-world practice.^22^ Predicted ASCVD risk was calculated for each participant at baseline, week 24, and trial end using predictors as measured at the respective time points. According to the ACC/AHA guidelines, risk scores were categorized as low (<5.0%), borderline (5.0%–7.5%), intermediate (7.5%–20.0%), or high (≥20.0%).^23^ Missing predictor values at all time points were not imputed; and when at least one predictor was missing for a participant at a given time point, an ASCVD risk was not calculated (i.e., missing) for that participant at that time point.

### Statistical analysis

Demographic and baseline clinical characteristics were compared between participants achieving BMI <25 kg/m^2^ at trial end versus those who did not. Chi-square tests and t-tests were conducted for categorical variables and continuous variables, respectively. A mixed model for repeated measures (MMRM) was used to compare percent change in predicted ASCVD risk from baseline to week 24 and trial end by achieved BMI at trial end (<25 kg/m^2^ or ≥25 kg/m^2^). The model included terms of achieved BMI group, time point, achieved-BMI-group-by-time-point interaction, and the following covariates at baseline: predicted ASCVD risk, sex, country, presence of at least one comorbidity (hypertension, dyslipidemia, obstructive sleep apnea, or cardiovascular disease), and BMI. Given the low overall predicted ASCVD risk, the 10-year predicted risk score and percent change from baseline were also assessed specifically within the intermediate-to-high baseline risk score group.

A series of subgroup analyses were performed, including by baseline overweight (BMI ≥25 to <30 kg/m^2^) or obesity (BMI >30 kg/m^2^) status, by baseline waist circumference categories (<100 cm for males or <95 cm for females; ≥100 cm to <110 cm for males or ≥95 cm to <105 cm for females; ≥110 cm for males or ≥105 cm for females), by baseline estimated glomerular filtration rate (eGFR, <90 mL/min/1.73 m^2^ or ≥90 mL/min/1.73 m^2^), by baseline non-alcoholic fatty liver disease (NAFLD; *note: NAFLD was collected in the SURMOUNT-1, -3, and -CN, but more recent clinical studies have shifted from the use of NAFLD toward metabolic dysfunction-associated fatty liver disease and metabolic dysfunction-associated steatotic liver disease*) status (with or without), and by baseline pre-diabetes status (with or without). In addition, three sensitivity analyses were performed. The first re-calculated the ASCVD risk using the PREVENT model,^24^ which is a race-free model more recently developed by the AHA. The second restricted the analysis population, in addition to the current inclusion and exclusion criteria, to participants aged 40–79 years to further align with the ACC/AHA PCE model development cohort. The third additionally adjusted for trial ID (SURMOUNT-1, -3, or -CN) in addition to the existing covariates in MMRM analysis.

In addition to the percent changes in predicted ASCVD risk, mean changes from baseline to week 24 and trial end in cardiometabolic risk factors (i.e., SBP, DBP, low-density lipoprotein [LDL] cholesterol, non-high-density lipoprotein [non-HDL] cholesterol, and urinary albumin-to-creatinine ratio [uACR]) were also compared by trial-end BMI using MMRM, adjusting for the same set of covariates described in main analysis except for baseline ASCVD risk, which was replaced with the corresponding cardiometabolic factor values at baseline. SAS 9.4 was used for all analyses.

### Role of funding source

This study was funded by Eli Lilly and Company. The study sponsor, Eli Lilly and Company, was involved in the study design, data collection, analysis, interpretation, and the writing of this report.

## Results

### Baseline characteristics

A total of 2691 participants were included for analysis, of whom 495 (18.4%) achieved BMI <25 kg/m^2^ at trial end (Supplementary Figure S4). Most (82.4%) participants were enrolled from the US and South American countries (Supplementary Table S5). The overall mean age was 45.0 (standard deviation [SD], 12.3) years, and 66.4% were female (Table 1). The proportion of participants who were randomized to tirzepatide 10 mg or 15 mg was significantly higher among those who achieved BMI <25 kg/m^2^ at trial end (79.8%) than in the BMI ≥25 kg/m^2^ group (46.4%, *P* < 0.001). Notably, participants achieving BMI <25 kg/m^2^ at trial end were also more frequently female (79.6% vs 63.4%, *P* < 0.001), Asian (22.6% vs 13.5%, *P* < 0.001), had lower BMI (32.3 [SD, 3.5] kg/m^2^ vs 38.2 [SD, 6.7] kg/m^2^, *P* < 0.001), smaller waist circumference (102.3 [SD, 10.1] cm vs 114.7 [SD, 15.0] cm, *P* < 0.001), higher serum LDL cholesterol (119.2 [SD, 34.0] mg/dL vs 113.6 [SD, 31.9] mg/dL, *P* < 0.001), and higher non-HDL cholesterol (147.1 [SD, 39.0] mg/dL vs 141.8 [SD, 36.1] mg/dL, *P* < 0.001).

**Table 1:** Demographic and baseline clinical characteristics of SURMOUNT-1, -3, and -CN participants categorized by achieved BMI.

Characteristics	BMI <25 kg/m^2^ group (n = 495)	BMI ≥25 kg/m^2^ group (n = 2196)	Total (N = 2691)	*P*-value
Trials				
SURMOUNT-1	374 (75.6)	1698 (77.3)	2072 (77.0)	
SURMOUNT-3	76 (15.4)	377 (17.2)	453 (16.8)	
SURMOUNT-CN	45 (9.1)	121 (5.5)	166 (6.2)	
Randomized treatment				<0.001
Placebo	9 (1.8)	728 (33.2)	737 (27.4)	
Tirzepatide 5 mg	91 (18.4)	450 (20.5)	541 (20.1)	
Tirzepatide 10/15 mg	395 (79.8)	1018 (46.4)	1413 (52.5)	
Age, years	45.0 ± 12.6	45.0 ± 12.2	45.0 ± 12.3	0.400
Sex (n, %)				<0.001
Female	394 (79.6)	1392 (63.4)	1786 (66.4)	
Race				<0.001
American Indian or Alaska native	26 (5.3)	179 (8.2)	205 (7.6)	
Asian	112 (22.6)	296 (13.5)	408 (15.2)	
White	332 (67.1)	1509 (68.7)	1841 (68.4)	
Black or African American	13 (2.6)	184 (8.4)	197 (7.3)	
Native Hawaiian or Other Pacific Islander	1 (0.2)	5 (0.2)	6 (0.2)	
Multiple	11 (2.2)	23 (1.0)	34 (1.3)	
Ethnicity				<0.001
Hispanic or Latino	190 (42.2)	1059 (51.0)	1249 (49.5)	
Not Hispanic or Latino	205 (45.6)	886 (42.7)	1091 (43.2)	
Not reported	55 (12.2)	130 (6.3)	185 (7.3)	
Current smoker				0.870
Yes	62 (12.5)	281 (12.8)	343 (12.7)	
Waist circumference, cm	102.3 ± 10.1	114.7 ± 15.0	112.4 ± 15.0	<0.001
BMI, kg/m^2^	32.3 ± 3.5	38.2 ± 6.7	37.2 ± 6.7	<0.001
SBP, mmHg	120.8 ± 12.8	123.1 ± 12.4	122.6 ± 12.5	<0.001
DBP, mmHg	78.8 ± 8.4	79.7 ± 8.4	79.5 ± 8.4	0.045
eGFR, mL/min/1.73 m^2^	96.8 ± 17.2	99.0 ± 17.6	98.6 ± 17.5	0.011
HbA1c, %	5.5 ± 0.4	5.5 ± 0.4	5.5 ± 0.4	<0.001
Serum Cholesterol, mg/dL	199.1 ± 40.4	189.7 ± 37.5	191.5 ± 38.2	<0.001
Serum non-HDL Cholesterol, mg/dL	147.1 ± 39.0	141.8 ± 36.1	142.8 ± 36.8	<0.001
Serum LDL Cholesterol, mg/dL	119.2 ± 34.0	113.6 ± 31.9	114.7 ± 32.3	<0.001
Baseline ASCVD risk score group				0.810
Low risk	410 (82.8)	1782 (81.1)	2192 (81.5)	
Borderline risk	6 (7.3)	174 (7.9)	210 (7.8)	
Intermediate risk	43 (8.7)	216 (9.8)	259 (9.6)	
High risk	6 (1.2)	24 (1.1)	30 (1.1)	

Data are n (%) or mean ± SD and include all patients in the efficacy analysis set with no baseline ASCVD and BMI <25 kg/m^2^.

ASCVD, atherosclerotic cardiovascular disease; BMI, body mass index; DBP, diastolic blood pressure; eGFR, estimated glomerular filtration rate; HbA1c, glycated hemoglobin; non-HDL, non-high-density lipoprotein; LDL, low-density lipoprotein; SBP, systolic blood pressure; SD, standard deviation.

### Percent changes in predicted 10-year ASCVD risk

**Fig. 1:**
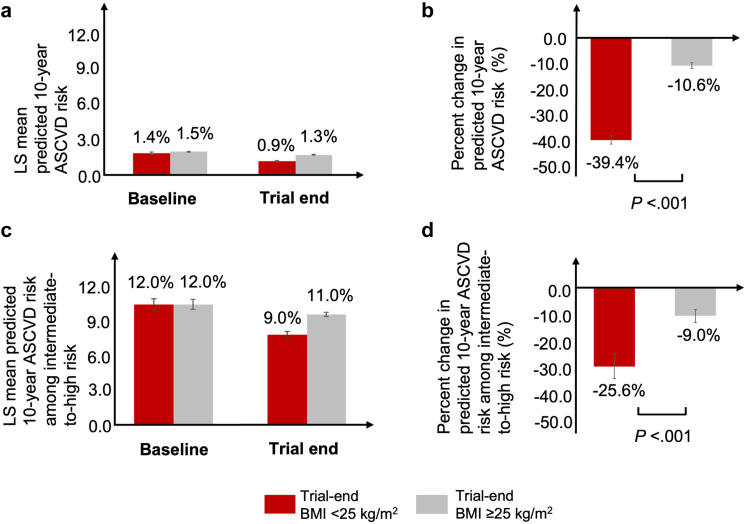
**Percent changes in predicted 10-year ASCVD risk by achieved BMI category.** a. Least squares (LS) mean predicted atherosclerotic cardiovascular disease (ASCVD) risk scores; b. Mean percent change in ASCVD risk from baseline to trial end; c. LS mean predicted ASCVD risk in participants with intermediate-to-high baseline risk; d. Mean percent change in ASCVD risk from baseline to trial end in participants with intermediate-to-high baseline risk. Error bar represents ±1 standard error. BMI, body mass index.

Baseline least squares (LS) mean predicted ASCVD risk score did not differ significantly between participants who achieved BMI <25 kg/m^2^ (1.4%, standard error [SE], 0.08%) and those who had BMI ≥25 kg/m^2^ at trial end (1.5% [SE, 0.04%]; *P* = 0.438) (Fig. 1a). At trial end, the LS mean predicted ASCVD risk score was 0.9% (SE, 0.02%) for BMI <25 kg/m^2^ group and 1.3% (SE, 0.02%) for BMI ≥25 kg/m^2^ group. The mean percent reduction from baseline to trial end in predicted ASCVD risk was significantly greater in participants achieving BMI <25 kg/m^2^ (39.4%, [SE, 1.6%]) compared with those with BMI ≥25 kg/m^2^ (10.6% [SE, 1.1%]; *P* < 0.001) (Fig. 1b). Among 289 participants with intermediate-to-high baseline ASCVD risk, whose mean age was 60.4 (SD, 9.8) years, the baseline LS mean predicted ASCVD risk was 12.0% in both BMI groups (BMI <25 kg/m^2^: SE, 0.6%; BMI ≥25 kg/m^2^: SE, 0.3%) (Fig. 1C), which decreased by 25.6% (SE, 4.0%) and 9.0% (SE, 2.1%) at trial end in the BMI <25 kg/m^2^ and BMI ≥25 kg/m^2^ groups, respectively. The percent ASCVD risk reduction from baseline to trial end also differed significantly between the two BMI groups (*P* < 0.001) (Fig. 1D).

### Subgroup and sensitivity analyses

**Fig. 2:**
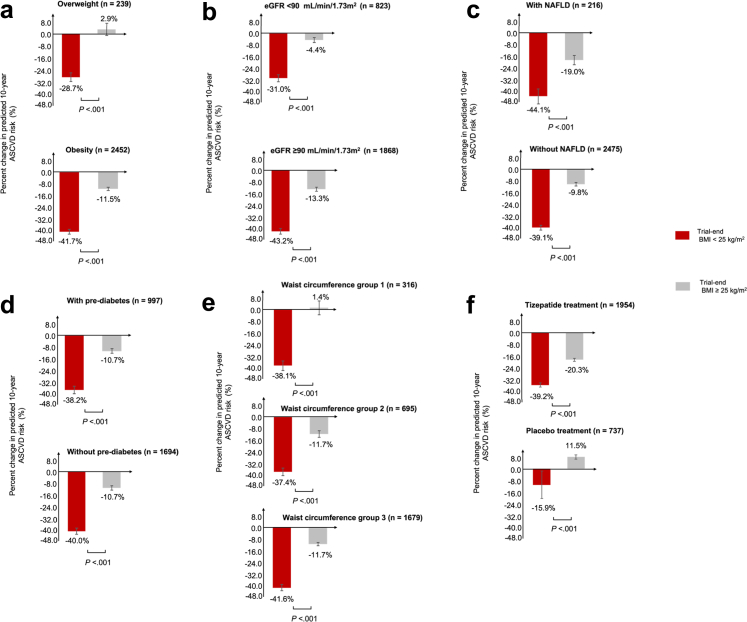
**Subgroup analyses of percent change in predicted 10-Year ASCVD risk by achieved BMI category.** a. Subgroup in baseline BMI (overweight: BMI ≥25 kg/m^2^ to <30 kg/m^2^; obesity: BMI >30 kg/m^2^); b. Subgroup in eGFR (<90 mL/min/1.73 m^2^; ≥90 mL/min/1.73 m^2^); c. Subgroup in NAFLD status (with; without); d. Subgroup in pre-diabetes status (with; without); e. Subgroup in baseline waist circumference (group 1: <100 cm for males or <95 cm for females; group 2: ≥100 cm to <110 cm for males or ≥95 cm to <105 cm for females; group 3: ≥110 cm for males or ≥105 cm for females); f. Assigned treatment (tirzepatide treatment; placebo treatment). ASCVD, atherosclerotic cardiovascular disease; eGFR, estimated glomerular filtration rate; NAFLD, non-alcoholic fatty liver disease.

Subgroup analyses consistently showed that participants achieving BMI <25 kg/m^2^ at trial end showed significantly greater percent reductions in the predicted ASCVD risk compared with those with BMI ≥25 kg/m^2^, regardless of their baseline obesity or overweight, waist circumference, eGFR level, NAFLD status, or prediabetes status (Fig. 2). Across the subgroups examined, the mean percent reduction in ASCVD risk appeared to be the greatest among patients with NAFLD. All three sensitivity analyses also yielded similar results (Supplementary Figure S6).

Because the assigned treatment was unbalanced between the two BMI groups, the percent risk changes were also explored within the tirzepatide- and placebo-treated participants, respectively. Among participants randomized to receive tirzepatide, baseline LS mean predicted ASCVD risks were 1.4% (SE, 0.09%) and 1.5% (SE, 0.05%) for those who achieved BMI <25 kg/m^2^ and those with BMI ≥25 kg/m^2^ at trial end, respectively. The mean percent risk reductions from baseline to trial end were 39.2% (SE, 1.6%) and 20.3% (SE, 1.2%) for the two subgroups, respectively (Fig. 2). Among participants randomized to placebo, those who achieved BMI <25 kg/m^2^ at trial end had a LS mean predicted risk of 0.8% (SE, 0.4%) at baseline and experienced a mean percent risk reduction of 15.9% (SE, 13.5%) at trial end; for those who had BMI ≥25 kg/m^2^ at trial end, the baseline risk was 1.4% (SE, 0.1%), and the predicted risk increased by 11.5% (SE, 2.1%) at trial end relative to baseline.

### Changes in cardiometabolic risk factors

**Fig. 3:**
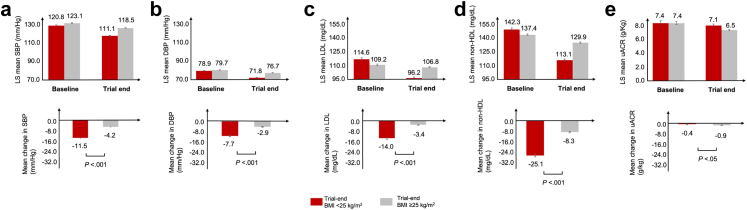
**Changes in cardiometabolic risk factors by achieved BMI category.** a. Least squares (LS) mean and change in systolic blood pressure (SBP); b. LS mean and change in diastolic blood pressure (DBP); c. LS mean and change in low-density lipoprotein (LDL); d. LS mean and change in non-high-density lipoprotein (non-HDL); e. LS mean and change in urinary albumin-to-creatinine ratio (uACR). Error bar represents ±1 standard error. BMI, body mass index.

Baseline and trial-end values of cardiometabolic risk factors, including SBP, DBP, LDL cholesterol, non-HDL cholesterol, and uACR, are shown in Fig. 3. The mean change from baseline to trial end was significantly greater in the BMI <25 kg/m^2^ group compared with the BMI ≥25 kg/m^2^ group for most risk factors excpet for uACR. Specifically, from baseline to trial end, participants who achieved BMI <25 kg/m^2^ at trial end experienced significantly greater reductions compared with the BMI ≥25 kg/m^2^ group in both SBP (11.5 [SE, 0.5] mmHg vs. 4.2 [SE, 0.2] mmHg; *P* < 0.001) and DBP (7.7 [SE, 0.4] mmHg vs. 2.9 [SE, 0.2] mmHg; *P* < 0.001). For lipids, the mean reduction in LDL cholesterol was significantly greater for the BMI <25 kg/m^2^ group (14.0 [SE, 1.1] mg/dL) than for the BMI ≥25 kg/m^2^ group (3.4 [SE, 0.6] mg/dL; *P* < 0.001), and similarly for non-HDL cholesterol (BMI <25 kg/m^2^: 25.1 [SE, 1.1] mg/dL vs BMI ≥25 kg/m^2^: 8.1 [SE, 0.6] mg/dL; *P* < 0.001). Mean reduction in uACR was significantly greater in the BMI ≥25 kg/m^2^ group (0.9 [SE, 0.1] g/kg) compared to the BMI <25 kg/m^2^ group (0.4 [SE, 0.2] g/kg; *P* = 0.028).

## Discussion

In this multi-country study cohort of participants with obesity or overweight without diabetes or ASCVD history, the study found that participants who achieved BMI <25 kg/m^2^ at trial end had significantly greater ASCVD risk improvement compared with those who had BMI ≥25 kg/m^2^ at trial end, in both the overall cohort and those with intermediate-to-high baseline predicted ASCVD risk. Moreover, participants who achieved BMI <25 kg/m^2^ also exhibited significantly greater improvements in cardiometabolic factors including SBP, DBP, LDL cholesterol, and non-HDL cholesterol. These findings suggested that, among individuals with obesity or overweight without diabetes, achieving BMI <25 kg/m^2^ via interventions might be linked to greater ASCVD benefits as compared to when BMI remained at higher levels.

Given the association between obesity and morbidity and mortality, weight management has a focus beyond weight itself, and prevention of ASCVD events should be one of its major long-term goals.^25^ While 5–15% weight reduction has remained the recommended target in mainstream weight management guidelines, it is worth noting that individuals with high BMI values at baseline may continue to have elevated, abnormal BMI following the initial 5–15% reduction. Weight reduction targets that are suitable for the long term is needed to optimize cardiovascular outcomes. Indeed, discussions reconsidering the percent weight reduction targets as well as more specific recommendations on the BMI target have been emerging.^26^ The latest European Society of Cardiology (ESC) guidelines, released in 2024, recommended targeting and maintaining a BMI of 20–25 kg/m^2^ to lower blood pressure and CVD risk.^27^ The current study provides indirect evidence on ASCVD benefits associated with targeting BMI <25 kg/m^2^, assisted by lifestyle interventions and/or pharmacotherapy, supporting the potential appropriateness of setting a long-term BMI target at below 25 kg/m^2^ among individuals with obesity or overweight without diabetes.

It is noteworthy that 98.2% of participants achieving BMI <25 kg/m^2^ in the current study were randomized to receive tirzepatide treatment. Weight targets like BMI <25 kg/m^2^ have become increasingly attainable with the emergence of such recent pharmacotherapies. Although lifestyle interventions remain the cornerstone of weight management, their efficacy in weight reduction can be limited, with most lifestyle interventions providing a weight reduction of <5%.^28^ A more substantial weight reduction, warranting more potent interventions such as bariatric surgery and pharmacotherapy, may be necessary to lead to a cardiovascular benefit.^28^^,^^29^ The nonrandomized, prospective Swedish Obese Subjects study (NCT01479452) showed a 23% weight reduction at 2 years following bariatric surgery.^30^ The STEP trials (NCT03548935, NCT03611582, NCT03548987, and NCT04074161) associated semaglutide, a GLP-1 receptor agonist, with a mean weight reduction of 14.9%–17.4% at week 68 among adults with overweight or obesity without diabetes.^31^ In SURMOUNT-1, -3, and -CN, tirzepatide was associated with a mean weight reduction between 13.6% and 22.5% at week 52 or 72 across different dosages.^17^, ^18^, ^19^ Cardiovascular benefits have been associated with these highly effective weight reduction interventions. Bariatric surgery was associated with a 53% reduction in cardiovascular death and 33% reduction in myocardial infarction or stroke, respectively, among adults with obesity over a median follow up of 14.7 years.^30^ The SELECT (NCT03574597) trial of non-diabetic patients with cardiovascular disease and overweight or obesity associated semaglutide with a 20% reduction in the risk of a composite endpoint of cardiovascular death, myocardial infarction, or stroke, over a mean follow up of nearly three years.^32^ Tirzepatide was associated with a 38% reduction in cardiovascular death or worsening heart failure over a median follow up of 2 years among patients with a heart failure history with preserved ejection fraction and obesity, as shown in the SUMMIT (NCT04847557) trial.^33^ In patients with diabetes, the SOUL (NCT03914326) and SURPASS-CVOT (NCT04255433) studies have associated semaglutide and tirzepatide, respectively, with cardiovascular benefits.^34^^,^^35^ Although the mechanism of the cardiovascular risk improvement has not been completely understood, the improvement is likely due to both the weight reduction and other metabolic effects of the weight reduction interventions, such as the anti-inflammatory effect of GIP and/or GLP-1 receptor agonists.^36^^,^^37^ In the current study, it is unlikely to distinguish whether the greater predicted ASCVD risk reduction observed in the BMI <25 kg/m^2^ group was attributable to the weight reduction to BMI <25 kg/m^2^ per se, to the treatment with tirzepatide, or to both.

Across subgroups examined in the current study, a greater reduction in predicted ASCVD risk was consistently observed in the BMI <25 kg/m^2^ group compared with the BMI ≥25 kg/m^2^ group. In particular, across the subgroups, the predicted ASCVD risk reduction from baseline to trial end appeared to be higher among participants with NAFLD, suggesting that NAFLD might be a factor modulating the cardiovascular benefits of weight reductions. Previous studies have demonstrated the association between NAFLD and the increased risk of hypertension and ASCVD.^38^ Obesity-induced inflammation, oxidative stress, mitochondrial dysfunction, gut dysbiosis, renin-angiotensin-aldosterone system overactivity, and endothelial dysfunction are further exacerbated by the development of NAFLD, ultimately accelerating ASCVD progression.^39^ Weight reduction has been shown to be associated with significant improvements in hepatic steatosis, non-alcoholic steatosis hepatitis, and fibrosis in patients with NAFLD.^40^^,^^41^ Further research is warranted to identify patient subgroups that may attain greater benefits from targeting stringent BMI control in the long term.

This study has several strengths. To our knowledge, this is the first study of a large, diverse sample exploring the association between achieving BMI <25 kg/m^2^ and 10-year predicted ASCVD risk among individuals with obesity or overweight without diabetes, with the ASCVD risk calculated using a well validated prediction model with a broad generalizability.^21^^,^^42^ In addition, the study results appeared to be robust as suggested by the subgroup analyses and sensitivity analyses.

The study also has limitations. First, the 10-year ASCVD risk was calculated using the prediction model ACC/AHA PCE, and no prediction model is perfectly accurate. However, this limitation is mitigated by the sensitivity analysis using another prediction model (the PREVENT), which led to consistent results, supporting the robustness of the results. Second, participants achieving BMI <25 kg/m^2^ at trial end differed from those with BMI ≥25 kg/m^2^ in baseline characteristics, likely leading to confounding bias. Nevertheless, baseline predicted ASCVD risk and BMI, among other baseline characteristics, were adjusted for in analyses. Third, the study grouping was based on BMI measured at trial end, whereas the outcome of interest was change in predicted ASCVD risk from baseline to trial end. The temporal sequence of the BMI <25 kg/m^2^ achievement and predicted risk change could not be established, preventing causal inference. With the current study providing early evidence on an association between BMI <25 kg/m^2^ achievement and a potential cardiovascular benefit, future studies based on long-term follow-up for hard outcomes are warranted. For example, the ongoing SURMOUNT-MMO (NCT05556512) trial randomized adults with obesity to tirzepatide or placebo, and the primary endpoint was a composite of all-cause death, myocardial infarction, stroke, coronary revascularization, or heart failure over up to 5 years. The availability of such a long-term follow-up monitoring weight and cardiovascular events will enable an assessment of the cardiovascular risk changes strictly after the achievement of BMI <25 kg/m^2^. Fourth, this study did not assess the long-term sustainability of BMI <25 kg/m^2^. Therefore, it remains unclear whether these cardioprotective benefits persist beyond the intervention, again highlighting the need for future studies with longer follow-up to evaluate the durability of both BMI <25 kg/m^2^ and its associated cardiovascular benefits. Fifth, this study focused on the achievement of BMI <25 kg/m^2^, although BMI has its own limitations as a measure of obesity such as not accounting for body composition variations (e.g., lean mass versus adipose tissue distribution). Finally, as mentioned earlier, this study was not able to differentiate the specific contributors to the observed reduction in ASCVD risk among participants achieving BMI <25 kg/m^2^, due to the collinearity between achievement of BMI <25 kg/m^2^ and the pharmacologic intervention.

BMI <25 kg/m^2^ was achieved in a considerable proportion of adults with obesity or overweight without diabetes receiving tirzepatide and was significantly associated with a decreased predicted 10-year ASCVD risk, compared with those remaining at elevated BMI levels (≥25 kg/m^2^). These findings suggest potential ASCVD benefits associated with targeting stringent BMI control in long-term weight management in a primary prevention setting. Future randomized controlled trials are warranted to evaluate the effectiveness, safety, and population-level applicability of stringent BMI control strategies, so as to inform future guidelines and practice.

## Contributors

All authors were involved in the drafting and revision of the manuscript and in the interpretation of the data. LG, HZ, and SjD were involved in study conceptualisation. LG, SD, WW, HZ, SjD, CL, and YY provided substantial contribution to study design. CL and YY were involved in formal analysis. AS, IR, and TDF were involved in data acquisition. YY and LG have directly accessed and verified the underlying data reported in the manuscript.

## Data sharing statement

Eli Lilly and Company provides access to all individual participant data collected during the trial, after anonymization, with the exception of pharmacokinetic or genetic data. Data are available to request six months after the indication studied has been approved in the US and EU and after primary publication acceptance, whichever is later. No expiration date of data requests is currently set once data are made available. Access is provided after a proposal has been approved by an independent review committee identified for this purpose and after receipt of a signed data sharing agreement. Data and documents, including the study protocol, statistical analysis plan, clinical study report, blank or annotated case report forms for the SURMOUNT-1 (NCT04184622), SURMOUNT-3 (NCT04657016), and SURMOUNT-CN (NCT05024032) studies will be provided in a secure data sharing environment. For details on submitting a request, see the instructions provided at http://www.vivli.org.

## Declaration of interests

HZ, SD, CL, YY, AS, IJ, and TDF are employees and mini shareholders of Eli Lilly and Company.

## Appendix A Supplementary data

Supplementary Material

Translated Abstract
