# Supplementary material for: Achieving BMI <25 kg/m2 was associated with reduced predicted risk of atherosclerotic cardiovascular disease in people with obesity or overweight on tirzepatide or placebo: a post hoc analysis of SURMOUNT-1, -3, and -CN
Source: eClinicalMedicine. 2025 Dec 27;91:103722. doi: 10.1016/j.eclinm.2025.103722 (PMC12796590; doi:10.1016/j.eclinm.2025.103722)
Supplement: Supplementary Material [file mmc1.docx]

**Supplementary materials**

**Achieving BMI <25 kg/m2 was Associated with Reduced Predicted Risk of Atherosclerotic Cardiovascular Disease in People with Obesity or Overweight on Tirzepatide or Placebo: A Post hoc Analysis of SURMOUNT-1, -3, and -CN**

Lixin Guo^1^, Shan Ding^1^, Weihao Wang^1^, Hanxi Zhang^2^, Shaojun Dai^2^, Chengwei Li^2^, Yuan Yuan^2^, Adam Stefanski^3^, Irina Jouravskaya^4^, Tammy D Forrester^3^

^1^ Department of Endocrinology, Beijing Hospital, National Center of Gerontology; Institute of Geriatric Medicine, Chinese Academy of Medical Sciences, P.R. China

^2^ Eli Lilly and Company, Suzhou, China

^3^ Eli Lilly and Company, Indianapolis, Indiana, USA

^4^ Eli Lilly and Company, Moscow, Russia

**Corresponding author:** Lixin Guo (email: [[**glx1218@163.com**](mailto:glx1218@163.com)](mailto:glx1218@163.com))

**Supplementary material**

**Supplementary Table S1** Ethics Review Boards for SURMOUNT-1 (NCT04184622)

**Supplementary Table S2** Ethics Review Boards for SURMOUNT-3 (NCT04657016)

**Supplementary Table S3** Ethics Review Boards for SURMOUNT-CN (NCT05024032)

**Supplementary Figure S4.** Patient selection.

**Supplementary Table S5**. Country/region distribution for included participants

**Supplementary Figure S6.** Sensitivity analyses of percent change in predicted 10-Year ASCVD risk.

**Supplementary Table S1. Ethics Review Boards for SURMOUNT-1 (NCT04184622)**

| **Site** | **Investigator Namea** | **ERB Name** | **ERB Address** |
| --- | --- | --- | --- |
| 100 |  | Advarra | 6100 Merriweather Dr., Suite 600, Columbia, MD 21044 |
| 101 |  | Advarra | 6100 Merriweather Dr., Suite 600, Columbia, MD 21044 |
|  |  |  |  |
|  |  |  |  |
| 102 |  | Advarra | 6100 Merriweather Dr., Suite 600, Columbia, MD 21044 |
| 103 |  | Advarra | 6100 Merriweather Dr., Suite 600, Columbia, MD 21044 |
| 104 |  | Advarra | 6100 Merriweather Dr., Suite 600, Columbia, MD 21044 |
| 105 |  | Advarra | 6100 Merriweather Dr., Suite 600, Columbia, MD 21044 |
| 106 |  | Advarra | 6100 Merriweather Dr., Suite 600, Columbia, MD 21044 |
|  |  |  |  |
| 107 |  | Advarra | 6100 Merriweather Dr., Suite 600, Columbia, MD 21044 |
| 108 |  | Advarra | 6100 Merriweather Dr., Suite 600, Columbia, MD 21044 |
| 109 |  | Advarra | 6100 Merriweather Dr., Suite 600, Columbia, MD 21044 |
| 110 |  | Advarra | 6100 Merriweather Dr., Suite 600, Columbia, MD 21044 |
| 111 |  | Advarra | 6100 Merriweather Dr., Suite 600, Columbia, MD 21044 |
|  |  |  |  |
| 112 |  | Advarra | 6100 Merriweather Dr., Suite 600, Columbia, MD 21044 |
| 113 |  | Advarra | 6100 Merriweather Dr., Suite 600, Columbia, MD 21044 |
| 114 |  | Advarra | 6100 Merriweather Dr., Suite 600, Columbia, MD 21044 |
| 115 |  | Advarra | 6100 Merriweather Dr., Suite 600, Columbia, MD 21044 |
| 116 |  | Advarra | 6100 Merriweather Dr., Suite 600, Columbia, MD 21044 |
| 117 |  | Advarra | 6100 Merriweather Dr., Suite 600, Columbia, MD 21044 |
| 118 |  | Advarra | 6100 Merriweather Dr., Suite 600, Columbia, MD 21044 |
| 119 |  | Advarra | 6100 Merriweather Dr., Suite 600, Columbia, MD 21044 |
| 120 |  | Advarra | 6100 Merriweather Dr., Suite 600, Columbia, MD 21044 |
|  |  |  |  |
| 121 |  | Advarra | 6100 Merriweather Dr., Suite 600, Columbia, MD 21044 |
|  |  |  |  |
| 122 |  | Advarra | 6100 Merriweather Dr., Suite 600, Columbia, MD 21044 |
| 123 |  | Advarra | 6100 Merriweather Dr., Suite 600, Columbia, MD 21044 |
| 124 |  | Advarra | 6100 Merriweather Dr., Suite 600, Columbia, MD 21044 |
| 125 |  | Advarra | 6100 Merriweather Dr., Suite 600, Columbia, MD 21044 |
| 126 |  | Advarra | 6100 Merriweather Dr., Suite 600, Columbia, MD 21044 |
| 127 |  | Advarra | 6100 Merriweather Dr., Suite 600, Columbia, MD 21044 |
| 128 |  | Advarra | 6100 Merriweather Dr., Suite 600, Columbia, MD 21044 |
| 129 |  | Advarra | 6100 Merriweather Dr., Suite 600, Columbia, MD 21044 |
| 130 |  | Advarra | 6100 Merriweather Dr., Suite 600, Columbia, MD 21044 |
| 131 |  | Advarra | 6100 Merriweather Dr., Suite 600, Columbia, MD 21044 |
| 132 |  | Advarra | 6100 Merriweather Dr., Suite 600, Columbia, MD 21044 |
| 133 |  | Advarra | 6100 Merriweather Dr., Suite 600, Columbia, MD 21044 |
| 134 |  | Advarra | 6100 Merriweather Dr., Suite 600, Columbia, MD 21044 |
|  |  |  |  |
|  |  |  |  |
| 135 |  | NYU School of Medicine Institutional Review Boards | 1 Park Avenue, 6^th^ Floor, Manhattan, New York County, NY 10016 |
| 136 |  | Advarra | 6100 Merriweather Dr., Suite 600, Columbia, MD 21044 |
| 137 |  | Advarra | 6100 Merriweather Dr., Suite 600, Columbia, MD 21044 |
| 138 |  | Advarra | 6100 Merriweather Dr., Suite 600, Columbia, MD 21044 |
|  |  |  |  |
| 139 |  | Advarra | 6100 Merriweather Dr., Suite 600, Columbia, MD 21044 |
| 141 |  | Advarra | 6100 Merriweather Dr., Suite 600, Columbia, MD 21044 |
| 142 |  | Advarra | 6100 Merriweather Dr., Suite 600, Columbia, MD 21044 |
| 143 |  | Advarra | 6100 Merriweather Dr., Suite 600, Columbia, MD 21044 |
| 144 |  | Advarra | 6100 Merriweather Dr., Suite 600, Columbia, MD 21044 |
|  |  |  |  |
| 145 |  | Advarra | 6100 Merriweather Dr., Suite 600, Columbia, MD 21044 |
| 146 |  | Advarra | 6100 Merriweather Dr., Suite 600, Columbia, MD 21044 |
| 147 |  | Advarra | 6100 Merriweather Dr., Suite 600, Columbia, MD 21044 |
|  |  |  |  |
| 148 |  | Advarra | 6100 Merriweather Dr., Suite 600, Columbia, MD 21044 |
| 149 |  | Advarra | 6100 Merriweather Dr., Suite 600, Columbia, MD 21044 |
| 150 |  | Advarra | 6100 Merriweather Dr., Suite 600, Columbia, MD 21044 |
| 151 |  | Advarra | 6100 Merriweather Dr., Suite 600, Columbia, MD 21044 |
| 152 |  | Advarra | 6100 Merriweather Dr., Suite 600, Columbia, MD 21044 |
| 153 |  | Advarra | 6100 Merriweather Dr., Suite 600, Columbia, MD 21044 |
| 154 |  | Advarra | 6100 Merriweather Dr., Suite 600, Columbia, MD 21044 |
| 155 |  | Advarra | 6100 Merriweather Dr., Suite 600, Columbia, MD 21044 |
|  |  |  |  |
| 156 |  | Advarra | 6100 Merriweather Dr., Suite 600, Columbia, MD 21044 |
|  |  |  |  |
| 157 |  | Weill Cornell Medical College | 1300 York Avenue, Box 89, New York, 10065 |
| 159 |  | Advarra | 6100 Merriweather Dr., Suite 600, Columbia, MD 21044 |
| 200 |  | The Fourth Affiliated Hospital of Harbin Medical University | Building 5, 11th Floor, No. 37, Yiyuan Street, Harbin, Nangang District, Harbin, 150001, China |
| 202 |  | Beijing Tsinghua Changgung Hospital | No. 168 Litang Road, Changping, Beijing, 102202, China |
| 203 |  | The First Affiliated Hospital of Xi'an Medical University | No. 48, Fenghao West Road, Lianhu District, Xi'an, Shanxi, China |
| 204 |  | Jinan Central Hospital | No. 105 Jiefang Road, Jinan, Shandong, 250013, China |
| 205 |  | Ningbo First Hospital | CCB Building 2206, No. 31, Guangji Street, Yuehu Street, Haishu District, Ningbo, Zhejiang, 315010, China |
| 208 |  | The Second Affiliated Hospital of Nanjing Medical University | 5^th^ Floor, Adult Fever Clinic, No. 121, Jiangjiayuan, Gulou District, Nanjing, Jiangsu, 210011, China |
| 301 |  | IEC CS at FSBI “National Medical Research Center of Cardiology named after E.I. Chazov” of the Ministry of Health of Russian Federation | 3rd Cherepkovskaya Street 15A, Moscow, 121552, Russian Federation |
| 302 |  | LEC at Saint-Petersburg SBHI “City Hospital #38 n.a. N.A. Semashko” | 7/2, Building A, Hospitalnaya Street, Pushkin, St. Petersburg, 196601, Russian Federation |
| 303 |  | LEC at FSBI “National Medical Research Center of Endocrinology” of the Ministry of Health of Russian Federation | 11, Dmitriya Ulyanova Street, Moscow, 117036, Russian Federation |
| 304 |  | EC at Regional Budgetary Healthcare Institution “Cardiology Dispensary” | Sheremetevskiy prospekt, 22, Ivanovo, 153012, Russian Federation |
| 305 |  | EC at Scientific Research Institution of Therapy and Preventive Medicine – Branch of the Institute of Cytology and Genetics of Siberian Branch of RAS | Borisa Bogatkova Street, 175/1, Novosibirsk, Novosibirsk Region, 630089, Russian Federation |
| 306 |  | LEC at BHI of Udmurtiya Republic “City Clinical Hospital #9” of Ministry of Healthcare of Udmurtiya Republic | Promyshlennaya Street, 52, Izhevsk, 426063, Russian Federation |
| 308 |  | LEC at FSAIE HE “Russian National Research Medical University named after N.I. Pirogov” | Ostrovityanova Street 1, Moscow, 117997, Russian Federation |
| 309 |  | IEC at Clinical Trials Center of FSAIE HE “Immanuel Kant Baltic Federal University” | 60, 9 Aprelya Street, Kaliningrad, 236035, Russian Federation |
| 400 |  | Comitê de Ética em Pesquisa em Seres Humanos do Hospital Pró | Rua Voluntários da Pátria 435, 8º andar Botafogo, Rio de Janeiro, Rio de Janeiro, 22270-005, Brazil |
| 401 |  | Comitê de Ética em Pesquisa do ISBEM - Instituto de Saúde e Bem-Estar da Mulher | 515 Rua Peixoto Gomide, cj 101 Jardim Paulista, Cj 101, São Paulo, São Paulo, 01409-001, Brazil |
| 402 |  | Comitê de Ética em Pesquisa do Centro Universitário FMABC | Av. Lauro Gomes, 2000, Prédio CEPES – 1º andar – sala 30 e 31, Santo André, São Paulo, 09060-870, Brazil |
|  |  |  |  |
| 403 |  | Comitê de Ética em Pesquisa ISBEM – Instituto de Saúde e Bem | Rua Peixoto Gomide, 515 - Cj 101 - Jardim Paulista - São Paulo, 01409 001, Brazil |
| 404 |  | Comitê de Ética em Pesquisa INVESTIGA – Instituto de Pesquis | Av. Romeu Tórtima, 739, Cidade Universitária Campinas, São Paulo, 13084-791, Brazil |
| 405 |  | Comissao de Etica para Analise Projetos de Pesquisa-CAPPesq | Rua Ovidio Pires de Campos 255 – 5ºandar, Predio da Administracao Cerqueira César, São Paulo, São Paulo, 05403-010, Brazil |
| 406 |  | Comitê de Ética em Pesquisa CIAS – Unimed Vitoria | Rua Marins Alvarino, 290, Itararé, Vitoria, Espirito Santo, 29046-660, Brazil |
| 407 |  | Comitê de Ética em Pesquisa INVESTIGA – Instituto de Pesquisa | Av. Romeu Tórtima, 739, Cidade Universitária Campinas, São Paulo, 13084-791, Brazil |
| 500 |  | Comité de Ética en Investigación Clínica (CEIC) | Paraná 755, 6° “A” y “B”, Ciudad Autónoma de Buenos Aires, Buenos Aires C1017AAP, Argentina |
| 501 |  | Comité de Ética del Centro de Osteopatías Médicas (CECOM) | Azcuenaga 1860, 1° Piso, CABA, Buenos Aires, C1128AAF, Argentina |
| 502 |  | Comité de Ética en Investigación Clínica (CEIC) | Paraná 755 6° “A” y “B”CABA, Buenos Aires, C1017AAP. Argentina |
| 503 |  | Centro de Investigaciones Metabólicas (CINME) | Presidente J.E Uriburu 754, 3° 12, Ciudad Autónoma de Buenos Aires, Buenos Aires, C1027AAP, Argentina |
| 504 |  | Comité de Ética en Investigación Clínica (CEIC) | Paraná 755 6° “A” y “B”CABA, Buenos Aires, C1017AAP, Argentina |
| 505 |  | Comité de Ética en Investigación Instituto de Investigaciones Clínicas | Av. Colón 3456, Mar del Plata, Buenos Aires, B7600FZN Argentina |
| 506 |  | Comité de Etica en Investigación Clínico Metabólico - CEIDICLIM v | Garibaldi 725, San Nicolás, Buenos Aires, B2900DPA Argentina |
| 508 |  | DIM Clínica Privada | Belgrano 136, Ramos Mejía, Buenos Aires, B1704ETD Argentina |
| 510 |  | Comité de Ética en Investigación Clínica | Paraná 755 6° “A” y “B”, Ciudad Autónoma de Buenos Aires, Buenos Aires, C1017AA, Argentina |
| 600 |  | Centro Especializado En Diabetes, Obesidad Y Prevención De Enfermedades Cardiovasculares | Calle 3 No. 7 Colonia Reforma Social, Mexico, D.F., 11650, Mexico |
| 601 |  | Hospital Hispano S.A. de C.V. | Pedro Moreno Número 934, Zona Centro, Guadalajara, Jalisco CP 44100, México |
| 602 |  | Hospital Universitario “Dr. Jose Eleuterio Gonzalez” | Av. Francisco I Madero y Av. Gonzalitos S/N Col. Mitras Centro, Monterrey, N.L., 64460 Mexico |
| 603 |  | Instituto Mexicano de Trasplantes S.C | Av. Alta Tensión 580-2, Col. Cantarranas, Cuernavaca, Morelos, México. CP 62448 |
| 604 |  | Unidad de Investigacion en Salud de Chihuahua, S.C. | Trasviña y Retes 1317, Col. San Felipe,  Chihuahua, Chihuahua, 31203, Mexico |
| 605 |  | Health Pharma Professional Research | Av. Insurgentes Sur 662 Piso 3, Col. Del Valle, Benito Juárez, CP. 03100, CDMX |
| 606 |  | Clinica Bajio CLINBA, S.C. | Calle Tomás Zavala, número 47, Col. Burócrata  Guanajuato, Guanajuato, 36256, Mexico |
| 607 |  | Hospital General de Culiacán, Dr. Bernardo J. Gastélum | Aldama y Nayarit S/N Col. General Antonio Rosales, Culiacán, Sinaloa, 80230, Mexico |
| 608 |  | Hospital General de Culiacán, Dr. Bernardo J. Gastélum | Aldama y Nayarit S/N Col. General Antonio Rosales, Culiacán, Sinaloa, 80230, Mexico |
| 609 |  | Accelerium S. de R.L. de C.V. | Modesto Arreola número 917 Oriente Col. Centro, Monterrey, Nuevo León, 64000 Mexico |
| 701 |  | Institutional Ethics Committee | CARE Hospitals, IP, 4th Floor, Room No-401, Road No. 1, Banjara Hills, Hyderabad 500 034 Telangana, India |
| 705 |  | Institutional Ethics Committee | Deenanath Mangeshkar Hospital & Research Center, Erandawane, Pune 411 004, Maharashtra, India |
| 708 |  | Institutional Ethics Committee | Department of Pharmacology, Grant Government Medical College and Sir JJ Group of Hospitals, Byculla, Mumbai 400 008, Maharashtra, India |
| 800 |  | Japan Conference of Clinical Research | 1-13-23, Minamiikebukuro, Toshima-ku, Tokyo, 171-0022, Japan |
| 801 |  | Tokyo-Eki Center-building Clinic | 3-3-14, Nihombashi, Chuo-ku, Tokyo, 103-0027, Japan |
| 802 |  | Osaka Pharmacology Clinical Research Hospital | 4-1-29, Miyahara, Yodogawa-ku, Osaka-City, Osaka, 532-0003, Japan |
| 803 |  | Medical Corporation Taifukukai, Tokyo Shinjuku Clinic | 2-46-3, Kabukicho, Shibjuku-ku, Tokyo, 160-0021, Japan |
| 804 |  | Tokyo-Eki Center-building Clinic | 3-3-14, Nihombashi, Chuo-ku, Tokyo, 103-0027, Japan |
| 900 |  | National Taiwan University Hospital Ethics Center Research Ethics Section | No. 7, Chung-Shan South Road, Zhongzheng District, Taipei City, 10048, Taiwan |
| 901 |  | China Medical University Hospital Research Ethics Committee | 2, Yude Road, Taichung (R.O.C.), 40447, Taiwan |
| 902 |  | Institutional Review Board, Chi-Mei Medical Center | 4F, 3rd Medical Building, No. 901, Zhonghua Road, Yungkang District, Tainan, 71004, Taiwan |
|  |  |  |  |
| 903 |  | The Institutional Review Board Chung Shan Medical University Hospital | 17F, Ruchuan Medical Building, No. 110, Sec. 1, Chien-Ko N. Road, South District, Taichung City, 40201, Taiwan |
| 904 |  | Institutional Review Board, National Cheng Kung University Hospital | No. 138, Sheng-Li Road, Tainan, 70457, Taiwan |
|  |  |  |  |

Abbreviation: ERB = ethics review board.

a Investigator names have been removed from the original table.

**Supplementary Table S2. Ethics Review Boards for SURMOUNT-3 (NCT04657016)**

| **Site** | **Investigator Name** | **ERB Name** | **ERB Address** |
| --- | --- | --- | --- |
| 22173 |  | Comité de Ética Independiente Consultorios Integrados CEICI | Comité de Ética Independiente Consultorios Integrados CEICI, Paraná 755 6ª A y B, CABA, CABA C1117ABK Argentina |
| 33649 |  | COMITE DE ETICA EN INVESTIGACION CLINICA CEIC | COMITE DE ETICA EN INVESTIGACION CLINICA CEIC Parana 755 6° A y B, Barrio Norte CABA, CABA C1017AAO, Argentina |
| 48517 |  | COMITÉ DE ÉTICA SAAVEDRA | COMITÉ DE ÉTICA SAAVEDRA, Av Ruiz Huidobro 4693, CABA  CABA 1430, Argentina |
| 54333 |  | Centro de Investigaciones Metabólicas (CINME) | Centro de Investigaciones Metabólicas (CINME), 754 Presidente José Evaristo Uriburu, Floor 3, Department 12 Balvanera, Comuna 3, CABA  CABA 1027, Argentina |
| 59465 |  | Comité de Ética Independiente Consultorios Integrados CEICI | Comité de Ética Independiente Consultorios Integrados CEICI, Paraná 755 6ª A y B, CABA, CABA, C1117ABK, Argentina |
| 66441 |  | Comité de Ética Independiente Consultorios Integrados CEICI | Comité de Ética Independiente Consultorios Integrados CEICI, Paraná 755 6ª A y B, CABA, CABA C1117ABK Argentina |
| 85301 |  | Comité de Ética Independiente Consultorios Integrados CEICI | Comité de Ética Independiente Consultorios Integrados CEICI, Paraná 755 6ª A y B, CABA, CABA C1117ABK Argentina |
| 86624 |  | CEMEDIAB | CEMEDIAB, Paraná 755 6to A y B, Barrio Norte Recoleta, Comuna 2 CABA CABA C1017AAO, Argentina |
| 87539 |  | Comité de Ética Independiente Consultorios Integrados CEICI | Comité de Ética Independiente Consultorios Integrados CEICI, Paraná 755 6ª A y B, CABA, CABA C1117ABK Argentina |
| 34871 |  | INVESTIGA - INSTITUTO DE PESQUISA | INVESTIGA - INSTITUTO DE PESQUISA, 739 Avenida Doutor Romeu Tortima, Jardim Santa Genebra II (Barao Geraldo), Campinas, São Paulo/Brazil 13084-791, Brazil |
| 35324 |  | Comitê de Ética em Pesquisa em Seres Humanos do Hospital Pró-Cardíaco | Comitê de Ética em Pesquisa em Seres Humanos do Hospital Pró-Cardíaco, Rua Voluntários da Pátria, 435, 2º floor, Rio de Janeiro, Rio de Janeiro/Brazil 20241-180, Brazil |
|  |  |  |  |
| 67400 |  | Comitê de Ética em Pesquisa Faculdade de Medicina do ABC | Comitê de Ética em Pesquisa Faculdade de Medicina do ABC, 2000 Avenida Lauro Gomes, Vila Sacadura Cabral, Santo André, São Paulo/ Brazil 09060-870, Brazil |
| 76126 |  | Comitê de Ética em Pesquisa em Seres Humanos - Centro Integrado de Atenção à Saúde - CIAS - UNIMED | Comitê de Ética em Pesquisa em Seres Humanos - Centro Integrado de Atenção à Saúde - CIAS - UNIMED, Rua Marins Alvarino, 290 - Itararé, Vitória, Espírito Santo/Brazil 29047-660, Brazil |
| 77166 |  | Instituto de Saúde e Bem-Estar da Mulher (ISBEM) | Instituto de Saúde e Bem-Estar da Mulher (ISBEM), Rua Manoel da Nóbrega, 784 Paraíso, São Paulo, São Paulo/Brazil 04001-002, Brazil |
| 90842 |  | Comitê de Ética em Pesquisa em Seres Humanos HC-UFPR | Comitê de Ética em Pesquisa em Seres Humanos HC-UFPR, General Carneiro 181, Curitiba, Paraná/Brazil 80060-900 Brazil |
| 97450 |  | Instituto de Saúde e Bem-Estar da Mulher (ISBEM) | Instituto de Saúde e Bem-Estar da Mulher (ISBEM), 515 Rua Peixoto Gomide cj 101 Jardim Paulista, São Paulo, Sao Paulo, São Paulo 01409-001, Brazil |
| 99845 |  | Universidade Positivo | Universidade Positivo, 5300 Rua Professor Pedro Viriato Parigot de Souza 2th floor, Curitiba, Paraná/Brazil 81270-020, Brazil |
| 12178 |  | Advarra Inc. | Advarra Inc., 6940 Columbia Gateway Drive, IRB Suite 110, Columbia  MD 21046, United States |
| 13455 |  | Advarra Inc. | Advarra Inc., 6940 Columbia Gateway Drive, IRB Suite 110, Columbia  MD 21046, United States |
| 14093 |  | Advarra Inc. | Advarra Inc., 6940 Columbia Gateway Drive, IRB Suite 110, Columbia  MD 21046, United States |
| 16181 |  | Advarra Inc. | Advarra Inc., 6940 Columbia Gateway Drive, IRB Suite 110, Columbia  MD 21046, United States |
|  |  |  |  |
| 20755 |  | Advarra Inc. | Advarra Inc., 6940 Columbia Gateway Drive, IRB Suite 110, Columbia  MD 21046, United States |
| 28324 |  | Advarra Inc. | Advarra Inc., 6940 Columbia Gateway Drive, IRB Suite 110, Columbia  MD 21046, United States |
| 28705 |  | Advarra Inc. | Advarra Inc., 6940 Columbia Gateway Drive, IRB Suite 110, Columbia  MD 21046, United States |
|  |  |  |  |
| 32915 |  | Advarra Inc. | Advarra Inc., 6940 Columbia Gateway Drive, IRB Suite 110, Columbia  MD 21046, United States |
| 38142 |  | Advarra Inc. | Advarra Inc., 6940 Columbia Gateway Drive, IRB Suite 110, Columbia  MD 21046, United States |
| 39281 |  | Advarra Inc. | Advarra Inc., 6940 Columbia Gateway Drive, IRB Suite 110, Columbia  MD 21046, United States |
| 41379 |  | Advarra Inc. | Advarra Inc., 6940 Columbia Gateway Drive, IRB Suite 110, Columbia  MD 21046, United States |
| 45115 |  | Advarra Inc. | Advarra Inc., 6940 Columbia Gateway Drive, IRB Suite 110, Columbia  MD 21046, United States |
| 46316 |  | Advarra Inc. | Advarra Inc., 6940 Columbia Gateway Drive, IRB Suite 110, Columbia  MD 21046, United States |
| 48652 |  | Vanderbilt Human Research Protections Program | Vanderbilt Human Research Protections Program, 3319 West End Avenue, Suite 600, Nashville, TN 37203, United States |
| 48920 |  | Advarra Inc. | Advarra Inc., 6940 Columbia Gateway Drive, IRB Suite 110, Columbia,  MD 21046, United States |
| 50701 |  | Advarra Inc. | Advarra Inc., 6940 Columbia Gateway Drive, IRB Suite 110, Columbia,  MD 21046, United States |
| 52006 |  | Advarra Inc. | Advarra Inc., 6940 Columbia Gateway Drive, IRB Suite 110, Columbia  MD 21046, United States |
| 54238 |  | Advarra Inc. | Advarra Inc., 6940 Columbia Gateway Drive, IRB Suite 110, Columbia  MD 21046, United States |
| 54900 |  | Advarra Inc. | Advarra Inc., 6940 Columbia Gateway Drive, IRB Suite 110, Columbia  MD 21046, United States |
| 55677 |  | Advarra Inc. | Advarra Inc., 6940 Columbia Gateway Drive, IRB Suite 110, Columbia  MD 21046, United States |
| 60382 |  | Advarra Inc. | Advarra Inc., 6940 Columbia Gateway Drive, IRB Suite 110, Columbia  MD 21046, United States |
| 61567 |  | Advarra Inc. | Advarra Inc., 6940 Columbia Gateway Drive, IRB Suite 110, Columbia  MD 21046, United States |
| 62852 |  | Advarra Inc. | Advarra Inc., 6940 Columbia Gateway Drive, IRB Suite 110, Columbia  MD 21046, United States |
| 65035 |  | Advarra Inc. | Advarra Inc., 6940 Columbia Gateway Drive, IRB Suite 110, Columbia  MD 21046, United States |
| 66515 |  | Advarra Inc. | Advarra Inc., 6940 Columbia Gateway Drive, IRB Suite 110, Columbia  MD 21046, United States |
| 68783 |  | Advarra Inc. | Advarra Inc., 6940 Columbia Gateway Drive, IRB Suite 110, Columbia  MD 21046, United States |
| 73122 |  | Advarra Inc. | Advarra Inc., 6940 Columbia Gateway Drive, IRB Suite 110, Columbia  MD 21046, United States |
| 76175 |  | Advarra Inc. | Advarra Inc., 6940 Columbia Gateway Drive, IRB Suite 110, Columbia  MD 21046, United States |
| 77815 |  | Advarra Inc. | Advarra Inc., 6940 Columbia Gateway Drive, IRB Suite 110, Columbia  MD 21046, United States |
|  |  |  |  |
| 78650 |  | Advarra Inc. | Advarra Inc., 6940 Columbia Gateway Drive, IRB Suite 110, Columbia  MD 21046, United States |
| 83064 |  | Advarra Inc. | Advarra Inc., 6940 Columbia Gateway Drive, IRB Suite 110, Columbia  MD 21046, United States |
| 83652 |  | Advarra Inc. | Advarra Inc., 6940 Columbia Gateway Drive, IRB Suite 110, Columbia  MD 21046, United States |
| 84547 |  | Advarra Inc. | Advarra Inc., 6940 Columbia Gateway Drive, IRB Suite 110, Columbia  MD 21046, United States |
| 89394 |  | Advarra Inc. | Advarra Inc., 6940 Columbia Gateway Drive, IRB Suite 110, Columbia  MD 21046, United States |
| 90694 |  | Advarra Inc. | Advarra Inc., 6940 Columbia Gateway Drive, IRB Suite 110, Columbia  MD 21046, United States |
| 91412 |  | Advarra Inc. | Advarra Inc., 6940 Columbia Gateway Drive, IRB Suite 110, Columbia  MD 21046, United States |
|  |  |  |  |
|  |  |  |  |
| 92027 |  | Advarra Inc. | Advarra Inc., 6940 Columbia Gateway Drive, IRB Suite 110, Columbia  MD 21046, United States |
| 92067 |  | Advarra Inc. | Advarra Inc., 6940 Columbia Gateway Drive, IRB Suite 110, Columbia  MD 21046, United States |
| 94902 |  | Advarra Inc. | Advarra Inc., 6940 Columbia Gateway Drive, IRB Suite 110, Columbia  MD 21046, United States |
| 95213 |  | Advarra Inc. | Advarra Inc., 6940 Columbia Gateway Drive, IRB Suite 110, Columbia  MD 21046, United States |
| 97504 |  | Advarra Inc. | Advarra Inc., 6940 Columbia Gateway Drive, IRB Suite 110, Columbia  MD 21046, United States |
| 97526 |  | Advarra Inc. | Advarra Inc., 6940 Columbia Gateway Drive, IRB Suite 110, Columbia  MD 21046, United States |
| 98434 |  | Advarra Inc. | Advarra Inc., 6940 Columbia Gateway Drive, IRB Suite 110, Columbia  MD 21046, United States |
|  |  |  |  |
| 98670 |  | Advarra Inc. | Advarra Inc., 6940 Columbia Gateway Drive, IRB Suite 110, Columbia  MD 21046, United States |
| 98895 |  | Advarra Inc. | Advarra Inc., 6940 Columbia Gateway Drive, IRB Suite 110, Columbia  MD 21046, United States |

Abbreviation: ERB = ethics review board.

a Investigator names have been removed from the original table.

**Supplementary Table S3. Ethics Review Boards for SURMOUNT-CN (NCT05024032)**

| 研究者姓名a/ 研究中心编号 | 伦理审查委员会的名称和地址 |
| --- | --- |
|  | 复旦大学附属中山医院医学伦理委员会  上海市徐汇区枫林路180号  中国，上海市 |
|  | 南京医科大学第二附属医院医学伦理委员会  南京市鼓楼区姜家园121号  中国，江苏省，南京市 |
|  | 南京市江宁医院伦理委员会  南京市江宁区湖山路169号  中国，江苏省，南京市 |
|  | 四川大学华西医院临床试验伦理审查委员会  成都市武侯区国学巷37号  中国，四川省，成都市 |
|  | 北京清华长庚医院医学伦理委员会  北京市昌平区立汤路168号  中国，北京市 |
|  | 秦皇岛市第一医院伦理委员会  秦皇岛市海港区文化路258号  中国，河北省，秦皇岛市 |
|  | 哈尔滨医科大学附属第四医院临床试验伦理委员会  哈尔滨市南岗区颐园街37号  中国，黑龙江省，哈尔滨市 |
|  | 深圳市第二人民医院药物临床试验伦理委员会  深圳市福田区笋岗路西路2008号  中国，广东省，深圳市 |
|  | 西安医学院第一附属医院人体研究伦理委员会  西安市莲湖区沣镐西路48号  中国，陕西省，西安市 |
|  | 常州市第二人民医院临床试验伦理委员会  常州市兴隆巷29号  中国，江苏省，常州市 |
|  | 无锡市人民医院临床试验伦理委员会  无锡市清扬路299号  中国，江苏省，无锡市 |
|  | 天津医科大学总医院药物伦理委员会  天津市和平区鞍山道154号  中国，天津市 |
|  | 河南科技大学第一附属医院医学伦理委员会  洛阳市涧西区景华路24号  中国，河南省，洛阳市 |
|  | 南华大学附属第一医院医学伦理委员会临床研究分伦理委员会  衡阳市船山路69号  中国，湖南省，衡阳市 |
|  | 上海市闵行区中心医院伦理委员会  上海市闵行区莘松路170号  中国，上海市 |
|  | 复旦大学附属中山医院青浦分院医学伦理委员会  上海市青浦区公园东路1158号  中国，上海市 |
|  | 四平市中心人民医院药物器械临床试验伦理委员会  四平市铁西区南迎宾街89号  中国，吉林省，四平市 |
|  | 宁波市第一医院伦理委员会  宁波市海曙区柳汀街59号  中国，浙江省，宁波市 |
|  | 宁波大学附属第一医院药械临床试验伦理委员会  宁波市海曙区柳汀街59号  中国，浙江省，宁波市 |
|  | 复旦大学附属上海市第五人民医院医学伦理委员会  上海市闵行区瑞丽路128号  中国，上海市 |
|  | 济南市中心医院医学伦理委员会  济南市历下区济南市中心医院解放路105号  中国，山东省，济南市 |
|  | 吉林大学第二医院医学伦理委员会  长春市南关区自强街218号  中国，吉林省，长春市 |
|  | 吉林大学第二医院医学伦理委员会  长春市南关区亚泰大街4110号  中国，吉林省，长春市 |
|  | 湖州市中心医院药物临床试验伦理委员会  湖州市吴兴区三环北路1558号  中国，浙江省，湖州市 |
|  | 重庆医科大学附属第一医院伦理委员会  重庆市渝中区袁家岗友谊路1号  中国，重庆市 |
|  | 郑州大学第二附属医院临床试验伦理委员会  郑州市金水区经八路纬三路规培中心  中国，河南省，郑州市 |
|  | 复旦大学附属华东医院伦理委员会  上海市静安区延安路221号  中国，上海市 |
|  | 南方医科大学珠江医院医学伦理委员会  广州市海珠区工业大道中253号  中国，广东省，广州市 |
|  | 杭州市第一人民医院伦理委员会  杭州市上城区浣纱路261号  中国，浙江省，杭州市 |
|  | 上海市中医医院伦理委员会  上海市静安区芷江中路274号  中国，上海市 |
|  | 南通大学附属医院伦理委员会  南通市西寺路20号  中国，江苏省，南通市 |

a Investigator names have been removed from the original table.


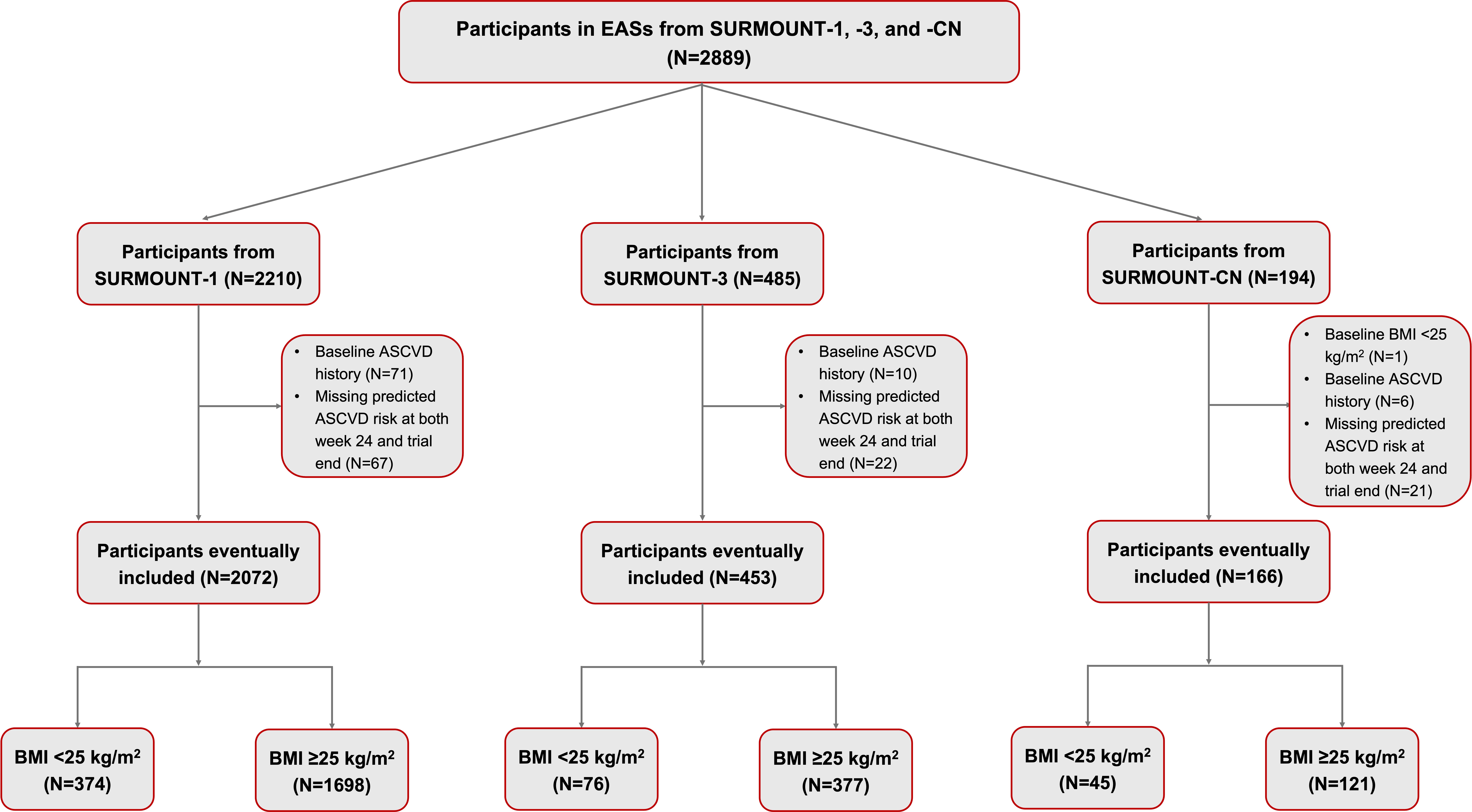


**Supplementary Figure S4. Patient selection**

ASCVD, atherosclerotic cardiovascular disease; BMI, body mass index; EASs, Efficacy Analysis Sets.

**Supplementary Table S5**. Country/region distribution for included participants

| **Country/Region** | **Frequency** | **Percent** |
| --- | --- | --- |
| United States | 1170 | 43.48 |
| Mexico | 397 | 14.75 |
| Argentina | 365 | 13.56 |
| Brazil | 286 | 10.63 |
| China | 189 | 7.02 |
| Japan | 109 | 4.05 |
| Russian Federation | 91 | 3.38 |
| Taiwan | 54 | 2.01 |
| India | 30 | 1.11 |
| Total | 2691 | 100.00 |


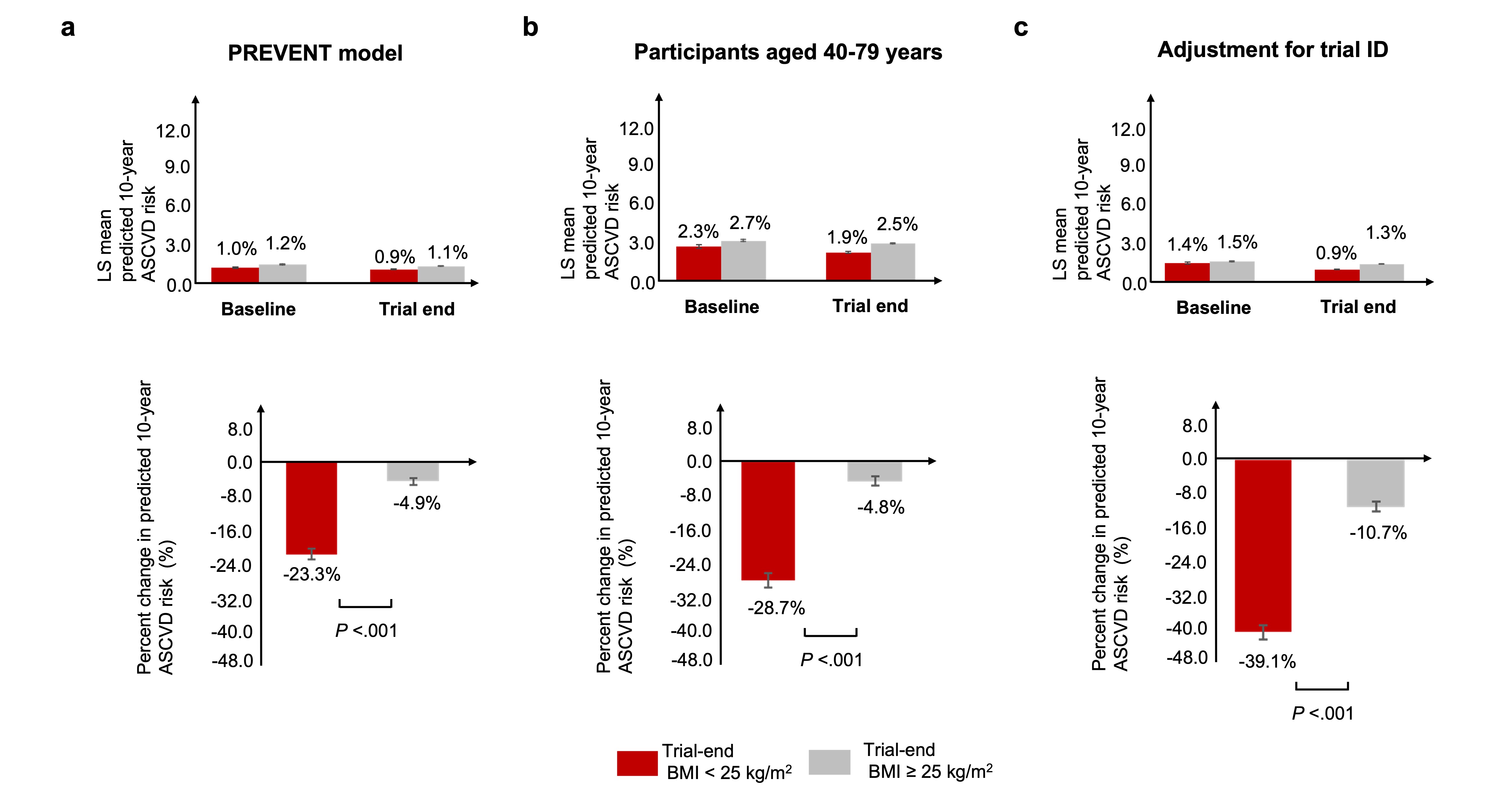


**Supplementary Figure S6. Sensitivity analyses of percent change in predicted 10-Year ASCVD risk**

a. Using the PREVENT model; b. Restriction to participants aged 40-79 years; c. Further adjustment for trial ID.

Error bar represents ± 1 standard error;

ASCVD, atherosclerotic cardiovascular disease; BMI, body mass index.
