# Supplementary material for: Achieving BMI <25 kg/m2 was associated with reduced predicted risk of atherosclerotic cardiovascular disease in people with obesity or overweight on tirzepatide or placebo: a post hoc analysis of SURMOUNT-1, -3, and -CN
Source: eClinicalMedicine. 2025 Dec 27;91:103722. doi: 10.1016/j.eclinm.2025.103722 (PMC12796590; doi:10.1016/j.eclinm.2025.103722)
Supplement: Translated Abstract [file mmc2.docx]

The following translations in Chinese were submitted by the authors and we reproduce them as supplied. They have not been peer reviewed. Our editorial processes have only been applied to the original abstract in English, which should serve as reference for this manuscript

**背景：** 有效的减重干预措施可能显著降低与肥胖相关的动脉粥样硬化性心血管疾病（ASCVD）风险。然而，关于最佳体重指数（BMI）目标与长期ASCVD风险之间关联的证据仍有限。

**方法：** 本研究分析了SURMOUNT-1（2019年12月–2022年4月；NCT04184622）、SURMOUNT-3（2021年3月–2023年4月；NCT04657016）和SURMOUNT-CN（2021年9月–2022年12月；NCT05024032）三项试验中接受替尔泊肽（tirzepatide）或安慰剂治疗的超重/肥胖受试者的个体级数据。使用美国心脏病学会/美国心脏协会的合并队列方程计算10年ASCVD风险。采用重复测量的混合模型分析，在试验结束时按达成的BMI分组（<25 kg/m²，≥25 kg/m²），比较ASCVD风险相较基线的百分比变化，模型包括达成的BMI分组、时间点、BMI分组与时间点的交互项，以及基线协变量。

**结果：** 共纳入2,691名参与者，其中495人（18.4%）在试验结束时达成BMI <25 kg/m²。与BMI ≥25 kg/m²组相比，BMI <25 kg/m²组中接受替尔泊肽治疗的比例显著更高（98.2% vs 66.8%），女性比例更高，且基线BMI均值更低（上述差异均P<0.001）。在校正基线协变量后，与BMI ≥25 kg/m²组相比，达成BMI <25 kg/m²的参与者的预测ASCVD风险较基线有显著更大的下降百分比（39.4% vs 10.6%，P<0.001）。在基线ASCVD风险为中高水平的参与者中，BMI <25 kg/m²组的风险下降百分比仍显著高于BMI ≥25 kg/m²组（25.6% vs 9.0%；P<0.001）。BMI <25 kg/m²组在血压和血脂方面也有显著更大的改善（P<0.001）。

**结论：** 达到BMI <25 kg/m²（本研究中主要通过使用替尔泊肽）与显著降低10年ASCVD风险相关。研究结果提示，在长期体重管理中，将BMI目标设定为<25 kg/m²可能带来潜在的心血管益处。

**资金来源：** 本研究由礼来公司（Eli Lilly and Company）资助。
